# Reviewed article: Silva PT, Toledo LV, Freitas BAC, Feitosa VA. Epidemiological profile, completeness of notifications, and factors associated with the adequacy of treatment for syphilis during pregnancy: a cross-sectional study, Ubá, 2018-2023. Epidemiol Serv Saude. 2026:35;e20250056

**DOI:** 10.1590/S2237-96222026v35e20250056.a

**Published:** 2026-03-20

**Authors:** Gabriel Pavinati

**Affiliations:** 1 Universidade Estadual de Maringá, Maringá, PR, Brazil

## Peer review A

## Questionnaire

Does the manuscript contain new and significant information to justify publication? Yes

Does the Abstract (Summary) clearly and accurately describe the content of the article? No

Is the problem significant and concisely stated? Yes

Are the methods described comprehensively? Yes

Are the interpretations and conclusions justified by the results? Yes

Is adequate reference made to other work in the field? Yes

## Manuscript Structure

Length of article is: Adequate

Number of tables is: Adequate

Number of figures is: Adequate

Please state any conflict(s) of interest that you have in relation to the review of this paper. None

Interest: Good

Quality: Good

Originality: Good

Overall: Good

Recommendation: Major Revision

## Comments

Resumo

Diz-se: “Calculou-se as taxas de incidência para regional”. E quanto a taxa de detecção?

Não apenas calculou o percentual de completude dos dados de notificação, eles foram categorizados.

Os resultados não estão contemplando o objetivo: não há menção do perfil descritivo analisado, conforme proposto no objetivo.

Na frase: “os dados de ambas as fichas apresentaram completude regular ou ruim para campos de preenchimento obrigatório e essencial”, é desconsiderado aqueles que tiveram bom preenchimento.

A conclusão e o objetivo precisam estar alinhados. Reflexões e hipóteses precisam ser descritas na seção de discussão.

Sugiro a substituição do descritor “vigilância em saúde pública” por “notificação de doenças”, visto que reflete um dos principais achados do trabalho.

Introdução

O primeiro parágrafo carece de citação.

Na apresentação do panorama epidemiológico, recomendo uma atualização. No terceiro parágrafo, por exemplo, são apresentados dados de 2016 (quase uma década atrás). Não há nada mais recente na literatura, visto que os padrões de incidência e mortalidade podem ter se alterado?

Informa-se que a “Organização Mundial da Saúde e seus parceiros lançaram uma iniciativa para eliminar a sífilis congênita”, porém não descreve qual. Dessa forma, a assertiva fica truncada. Detalhar.

Após informar: “considerando aceitável a incidência de até 0,5 casos por mil nascidos vivos (6)”, explicitar a incidência atual no Brasil, cenário da pesquisa [uma sugestão é transplantar o parágrafo já escrito - O país tem se confrontado com números distantes das metas pactuadas internacionalmente para eliminação desta infecção. Em 2023, apresentou incidência de 34,0 casos de sífilis gestacional e 9,9 casos de sífilis congênita por mil nascidos vivos (8)]. Após a finalização desse parágrafo, fazer um link melhor com o parágrafo seguinte, talvez, discorrendo que a notificação é uma estratégia de vigilância que auxilia na tomada de decisão.

A frase: “A maioria das gestantes foi diagnosticada em tempo oportuno, mas a qualidade da assistência pré-natal pode ser questionada (10)”, foi suportada com a menção a um boletim epidemiológico da secretaria de saúde local. Acredito que os autores precisam explicitar melhor o juízo de valor que infere a qualidade do serviço. Quais são as razões? Há algum dado em particular que os pesquisadores atribuem a isso? Para quem não conhece a realidade local, a afirmação fica subjetiva e frágil.

No objetivo, sugiro incluir o período de estudo.

Método

Pontuo uma reflexão aos autores em relação ao tipo de estudo: na forma atual, está definido como pesquisa transversal analítica, no entanto, me parece que o foco é muito mais descritivo-exploratório do que de associação (exposição-desfecho). Apenas a inclusão de um teste de hipótese por si só não categoriza o estudo como analítico. Como os autores assumem está questão?

A inclusão do “tratamento não realizado”, presente no campo “esquema de tratamento prescrita à gestante”, poderia ser um viés interpretativo? Questiono, pois, por vezes, a não realização do tratamento pode se dar por não adesão do usuário e não por não prescrição. Qual a perspectiva dos autores em relação a isso? Não seria interessante a “não realização do tratamento” ser uma terceira categoria?

Diz-se: “Quando os pressupostos não foram atendidos, utilizou-se os testes Exato de Fisher e Mann-Whitney”. Estabelecer quais.

Seria interessante estarem previstas cada uma das variáveis analisadas logo na seção metodológica. Sugiro a inclusão.

Para construção da variável ‘prescrição adequada’, a análise da prescrição segundo classificação clínica da sífilis foi feita de maneira manual pelos pesquisadores? Houve dupla checagem? Descrever melhor essa etapa, especialmente por se tratar da construção da variável dependente do estudo.

Resultados

Menciona-se Figura Suplementar 1, porém não está disponível para avaliação.

Nas notas da tabela 1, especificar quais variáveis foram expressas em frequência e quais foram por meio de mediana e intervalo interquartil (e.g., variáveis numéricas foram expressas em mediana). Na forma atual, a interpretação é pouco intuitiva e didática. Considerar o mesmo para as demais tabelas.

Acredito que seja relevante justificar a junção das categorias ‘preta’ e ‘parda’ e posterior denominação em ‘negra’. Há correntes teóricas que se debruçam nisso e fundamentam tal decisão?

No terceiro parágrafo dos resultados, quando apresentados os resultados numéricos (i.e., ‘46,7% vs. 23,6%’) recomendo que seja informado apenas a porcentagem referente à prescrição inadequada daquela categoria. A apresentação de ambos os valores torna a leitura confusa. Além disso, a preposição ‘vs’ abreviada é bastante informal e deve ser evitada. Isso também vale para o resumo.

Na Tabela 2, a categoria de referência para a análise bivariada não está determinada. Rever.

Considerando que para sífilis materna houve a proposição de uma análise bivariada para investigar as variáveis associadas.

Por que a informação de tratamento da parceria sexual não foi reportada na tabela?

Discussão

Como o estudo centra-se no cuidado materno-infantil e na qualidade da assistência prestada, acredito ser indispensável fazer menção as políticas de saúde vigentes que norteiam esse cuidado, sobretudo pensando no escopo da revista. Recomendo fazer alusão a Rede Cegonha e a sua operacionalização no cenário local, fazendo uma correlação com os achados.

Ainda, a discussão carece de um parágrafo que destaque a provável influência da covid nos resultados. Os autores analisaram dados entre 2018 e 2023, ou seja, 2020, 2021 e 2022 (1/2 do período) foi fortemente influenciado pela pandemia. Houve subdiagnóstico, subnotificação, subpreenchimento, muito pela alta demanda de atendimentos e notificações da covid-10. Penso que isso precisa ser destacado na discussão.

Sugiro um parágrafo que destaque quais são as principais limitações da pesquisa e como elas interferem nos achados e interpretações.

Referências

Dez (10) referências são anteriores a cinco anos. Sugiro atualizar as que são possíveis.

